# Statistical optimization of tretinoin-loaded penetration-enhancer vesicles (PEV) for topical delivery

**DOI:** 10.1186/s40199-016-0142-0

**Published:** 2016-02-29

**Authors:** Neda Bavarsad, Abbas Akhgari, Somayeh Seifmanesh, Anayatollah Salimi, Annahita Rezaie

**Affiliations:** Nanotechnology Research Center, Ahvaz Jundishapur University of Medical Sciences, Ahvaz, Iran; Department of Pharmaceutics, School of Pharmacy, Ahvaz Jundishapur University of Medical Sciences, Ahvaz, Iran; Targeted Drug Delivery Research Center, School of Pharmacy, Mashhad University of Medical Sciences, Mashhad, Iran; Department of Pathobiology, Faculty of Veterinary Medicine, Shahid Chamran University of Ahvaz, Ahvaz, Iran

**Keywords:** Statistical optimization, Tretinoin, Liposomal formulation, Topical delivery

## Abstract

**Background:**

The aim of this study was to develop and optimize deformable liposome for topical delivery of tretinoin.

**Methods:**

Liposomal formulations were designed based on the full factorial design and prepared by fusion method. The influence of different ratio of soy phosphatidylcholine and transcutol (independent variables) on incorporation efficiency and drug release in 15 min and 24 h (responses) from liposomal formulations was evaluated. Liposomes were characterized for their vesicle size and Differential Scanning Calorimetry (DSC) was used to investigate changes in their thermal behavior. The penetration and retention of drug was determined using mouse skin. Also skin histology study was performed.

**Results:**

Particle size of all formulations was smaller than 20 nm. Incorporation efficiency of liposomes was 79–93 %. Formulation F7 (25:5) showed maximum drug release. Optimum formulations were selected based on the contour plots resulted by statistical equations of drug release in 15 min and 24 h. Solubility properties of transcutol led to higher skin penetration for optimum formulations compared to tretinoin cream. There was no significant difference between the amount of drug retained in the skin by applying optimum formulations and cream. Histopatological investigation suggested optimum formulations could decrease the adverse effect of tretinoin in liposome compared to conventional cream.

**Conclusion:**

According to the results of the study, it is concluded that deformable liposome containing transcutol may be successfully used for dermal delivery of tretinoin.

## Background

Tretinoin or all-trans retinoic acid in topical form is commonly used for the treatment of various skin problems like acne, photoaging and psoriasis. Furthermore, it has other functions such as sebum production, collagen synthesis and regulating growth and differentiation of epithelial cells [1]. Reduction the size and the number of comedones are considered to be the main effect of tretinoin in the treatment of acne [2]. However, it has several negative points even in topical use such as very low water solubility, skin irritation and high instability in the presence of air, light and heat. Furthermore, local irritation such as erythema, peeling and burning at the application site and increased susceptibility to sunlight are its side effects [3].

Liposomes are spherical-shaped carriers which has an internal aqueous portion surrounded by one or multiple concentric lipidic bilayers. Liposomes are used as carriers for both lipophilic and water soluble molecules. Hydrophilic substances are encapsulated in the interior aqueous portions whereas lipophilic substances are entrapped within lipid bilayers [4].

Incorporation of tretinoin in nanostructure systems such as liposomes may lead to decrease the adverse effects and protect this molecule against degradation [5–8].

Liposomes were first used as topical therapy by Mezei and Gulasekharam in 1980. The introduction of liposomes as skin drug delivery systems, initially promoted for localized effects with minimal systemic delivery. Reduction of vesicle size improves drug deposition into deeper strata. Recent advances and alteration in the composition and structure of vesicles result in vesicles with tailored properties [9].

The first generations of elastic vesicles are deformable liposomes (Transfersomes®) which consist of phospholipids and surfactant as an edge activator. Surfactant destabilizes lipid bilayers and increases deformability of the vesicles. Ethosome is another type of elastic vesicle which is composed of phospholipid, ethanol and water [10]. Recently, a novel family of liposomes which named the Penetration Enhancer-containing Vesicles (PEVs) is described for enhanced (trans)-dermal drug delivery [11]. They mainly consist of phospholipids and penetration enhancers with hydrosoluble glycols such as diethylene glycol mono ethyl ether or propylene glycol. Transient reduction of the stratum corneum barrier function and improvement in vesicular bilayer fluidity are two main functions of the PEVs [12].

The aim of this study was to prepare and evaluate deformable liposome using soy phosphatidylcholine and transcutol (Diethylene glycol monoethyl ether) for topical delivery of tretinoin. Also optimization of formulation was performed in order to obtain suitable dermal delivery system for tretinoin.

## Methods

### Materials

Tretinoin was purchased from Sepidaj (Iran). Soy phosphatidylcholine (phospholipon 85G®) was obtained from lipoid (Germany). Cholesterol and HEPES (4-(2-Hydroxyethyl) 1-piperazine ethanesulfonic acid) were purchased from Sigma)Germany). Diethylene glycol monoethyle ether (Transcutol®) received as a gift from Gattefosse (France). Propyl paraben, methyl paraben, propylene glycol and vitamin E were obtained from Merck (Germany). Tretinoin cream 0.05 % was purchased from the pharmacy and its base cream was provided as a gift by Iran Daru (Iran).

### Animals

Female NMRI mice 7–9 weeks old were obtained from laboratory Animals Care and Breeding Center of Ahvaz Jundishapur University of Medical Sciences (Ahvaz, Iran). The experiments were conducted in full compliance with regulatory principles of ethics committee of Ahvaz Jundishapur University of Medical Sciences.

### Experimental design

A full factorial 3^2^ design was used for optimization procedure. The studied independent variables were amount of soy phosphatidylcholine (SPC) (X_1_) and amount of transcutol (X_2_) in formulation. Types and levels of the independent variables are listed in Table 1. Dependent variables (responses) were percent of incorporation efficiency (Y_1_), percent of drug release in 15 min (Y_2_) and percent of drug release in 24 h (Y_3_). The resulted formulations are illustrated in Table 2.

**Table 1:** Independent variables: Types and Levels

Variables	Levels		
X_1_: amount of SPC (% wt)	15	20	25
X_2_: amount of transcutol (% wt)	5	10	15

**Table 2:** Composition of experimental formulations (runs)

Run	X_1_: SPC (% wt)	X_2_: transcutol (% wt)
1	15	5
2	15	10
3	15	15
4	20	5
5	20	10
6	20	15
7	25	5
8	25	10
9	25	15

### Preparation of deformable liposomes

Deformable liposomes were prepared by the fusion method. Briefly, the lipid components consisted of SPC (15–20–25 % wt), transcutol (5–10–15 % wt), tretinoin) 0.05 % wt), cholesterol (2 % wt), propylene glycol (3 % wt), vitamin E (0.3 % wt), metyl paraben (0.1 % wt), and propyl paraben (0.02 % wt); these components were melted at about 75 °C. HEPES buffer (10 mM, pH 5) was heated separately and was added up to 100 % to the previously heated melted lipids, and the mixture was homogenized with a homogenizer (Ultra-Turrax IKA T25) for 5 min at 12,000 rpm and allow it to cool down to room temperature [13].

### Characterization of the liposomes

#### Particle size measurement

The particle size of the samples were measured in triplicate by laser light scattering (Scatterscope 1, Qudix, South Korea). Samples were diluted in HEPES buffer to a suitable concentration (0.2 g formulation in 1 ml HEPES buffer).

#### Incorporation efficiency

Incorporation efficiency of liposomes was determined indirectly. Certain amounts of liposomal dispersions were centrifuged (VS-35SMTI, Korea) at 20,000 rpm for 25 min at 25 °C. The supernatant was collected and analyzed at 362 nm using UV spectrophotometer (Biowave II, Biochrom, England) [14]. The percent of incorporation efficiency of drug was calculated by the following formula:

$$ \mathrm{E}\mathrm{E}\% = \left[\left(\mathrm{amount}\ \mathrm{of}\ \mathrm{in}\mathrm{itial}\ \mathrm{drug}\ \hbox{--}\ \mathrm{amount}\ \mathrm{of}\ \mathrm{free}\ \mathrm{drug}\ \mathrm{in}\ \mathrm{supernatant}\right)/\mathrm{amount}\ \mathrm{of}\ \mathrm{in}\mathrm{itial}\ \mathrm{drug}\right] \times 100 $$

### In vitro drug release

Drug release studies were performed using dialysis membrane method. Dialysis membranes were soaked before use in distilled water for 20 h. 1 g of formulation was placed in a dialysis membrane and both ends were closed. The membrane was float in a beaker containing 150 ml phosphate buffer (pH 7.4) and methanol (2:1 v/v); and stirred at 200 rpm at 37 °C. 1 ml of receiver medium was removed at 0.25, 0.5, 0.75, 1, 1.5, 2, 4, 6, 8 and 24 h and same volume of the fresh medium was replaced. The collected samples were analyzed for their tretinoin content. The derived concentration values were corrected by using the equation (1):

1 $$ \mathrm{M}\mathrm{t}\ \left(\mathrm{n}\right)=\mathrm{V}\mathrm{r}\times \mathrm{C}\mathrm{n}+\mathrm{V}\mathrm{s}\times \sum \mathrm{C}\mathrm{m} $$

Where Mt(n) is the current cumulative mass of drug transported across the membrane at time t, n is the number (times) of sampling, Cn is the current concentration in the receiver medium, ∑Cm is the summed total of the previously measured concentrations, Vr is the volume of the receiver medium, and Vs corresponds to the volume of the sample removed for analysis [15, 16].

### Viscosity

Viscosity of selected formulations were measured by Brookfield viscometer (DV II + Pro, US) at 10 rpm and 25 °C using spindle 64.

### In vitro skin penetration and retention

In vitro skin penetration studies were performed using jacketed Franz cells with a receiver medium of 25 ml Phosphate buffer (pH 7.4) and methanol (2:1 v/v) at 37 °C and surface area of 4.84 cm^2^. The dorsal skin of mouse was shaved with electric clippers one day before the experiment. A suitable size of full-thickness skin of mouse was cut and clamped between the donor and receiver compartment of Franz cell with the stratum corneum side facing upward. The skin samples were initially left in the Franz cells for 30 min in order to facilitate hydration. Subsequently, 1 g of the optimum formulations and tretinoin cream were placed onto the skin surface. 1 ml of receiver solution was removed at 0.25, 0.5, 0.75, 1, 1.5, 2, 4, 6 and 8 h and same volume of the fresh medium was replaced. The collected samples were analyzed for their tretinoin content. The derived concentration values were corrected according to equation (1).

For the determination of the amount of the drug retained in the skin, at the end of the experiment, the amount of the formulation remaining on the surface of the skin was collected and assayed for tretinoin. The amount of tretinoin retained in the skin was then calculated by subtracting the sum of the amount of tretinoin that remained on the surface and the amount of tretinoin that was released (penetrated through the skin) from the whole applied amount [13].

The cumulative amount of drug permeated was plotted against time. The steady-state permeation rate (J_ss_) was calculated by divided the slop of the linear portion of the plot on the exposed surface area of the skin. Lag time was determined from the x-intercept of the linear portion of the plot [17].

### Histological evaluation

For skin histological study the dorsal side of the mouse was shaved with electric clipper. The skin was cut and mounted between donor and receiver compartment of the jacketed Franz cell whit stratum corneum side facing upward. 1 g of optimum formulations and tretinoin cream were placed on the skin. After 38 h the excessive formulation and cream were removed and the skin cleaned with cotton soaked in a phosphate buffer solution (pH 7.4). The treated skins were fixed in 10 % formalin solution and embedded in paraffin wax. Then samples were cut in 5 μm thick sections by using microtome and conventionally stained with haematoxylin and eosin (H&E). Finally the samples were examined by light microscope (Olympus, BH-2, Japan) [18].

### Differential scanning calorimetry (DSC)

DSC thermograms of SPC, cholesterol, transcutol and tretinoin were recorded on a differential scanning calorimeter (Mettler Toledo, DSC-1, Switzerland). Thermograms of both blank and tretinoin loaded liposomes were recorded individually. Certain amount of sample was placed in aluminum pan and scanned from 20 to 200 °C by scanning rate of 10 °C/min.

### Stability study

Optimum formulations were stored at refrigerate temperature (4 °C) for 3 months. After 1 and3 months, the particle size and drug incorporation efficiency of the formulations were measured. The results were compared with the initial size and drug incorporation efficiency of formulations [19].

### Statistical analysis

All experiments were repeated three times and expressed as the mean ± standard deviation. One way analysis of variance (ANOVA) followed by multiple comparisons Tukey test was used to substantiate statistical differences between groups. Results with *P* < 0.05 were considered to be significant.

The effects of independent variables (X) on the dependent variables (Y) were modeled using a polynomial second order equation as followed:

$$ \mathrm{Y} = \mathrm{c} + {\mathrm{b}}_1{\mathrm{X}}_1 + {\mathrm{b}}_2{\mathrm{X}}_2 + {\mathrm{b}}_3{{\mathrm{X}}_1}^2 + {\mathrm{b}}_4{{\mathrm{X}}_2}^2 + {\mathrm{b}}_5{\mathrm{X}}_1{\mathrm{X}}_2 $$

The modeling was performed using SPSS (Version 16.0) with a backward, stepwise linear regression technique and significant expressions (*P* < 0.05) were selected for the final equations. Response surface plots and contour plots resulting from equations obtained by Statgraphics version Centurion XVI.

## Results and discussion

In an attempt to obtain suitable formulation containing tretinoin for dermal delivery, deformable liposomes prepared with SPC and transcutol.

Transcutol (diethylene glycol monoethylether) is a non-toxic, biocompatible with skin, penetration enhancer which is miscible in polar and non polar solvents [20]. Effect of transcutol on the lipid organizational structure of human stratum corneum was evaluated by Moghadam et al. [21]. Their results of x-ray scattering study showed transcutol caused a slight disordering effect in the stratum corneum membrane and increasing its fluidity.

In this study the fusion method was used to prepare the topical liposomal formulations. This method is simple, efficient, and reproducible. The method is free of organic solvents like chloroform; and yields homogeneous liposomes with high incorporation efficiencies. Furthermore, liposomes prepared by fusion method showed enough viscosity that they could be applied directly on the skin without the need for the liposomal formulation to be mixed with other bases [9].

### Characterization of the liposomes

#### Particle size

The mean particle size of the liposomal formulations was shown in Table 3. ANOVA analysis showed statistical significant differences between F2 and F1, F8, F9 (*P* < 0.05) and also, F4 and F9 (*P* < 0.05). Different concentrations of phospholipid and transcutol showed no effect on particle size of liposomal formulations.

**Table 3:** Particle size (nm) and Incorporation efficiency (%) of liposomal formulations (mean ± SD, *n* = 3)

Formulation	Average size	Incorporation (%)
1	8.82 ± 0.95	85.51 ± 0.15
2	16.96 ± 3.69	80.88 ± 0.19
3	10.94 ± 1.84	79.11 ± 0.19
4	15.09 ± 4.62	89.55 ± 0.19
5	9.59 ± 2.99	91.33 ± 0.57
6	12.8 ± 1.65	89.88 ± 0.19
7	10.21 ± 1.85	93.22 ± 0.69
8	7.70 ± 2.30	93.88 ± 0.19
9	7.12 ± 0.74	89.99 ± 0.33

#### Incorporation efficiency

The incorporation efficiency of formulations was in range of 79 to 93 % (Table 3). F8 showed maximum percent of incorporation. There were no statistical significant differences among F4, F6, F9 (*P* > 0.05) and also, between F7 and F8 (*P* > 0.05).

Mathematical relationships were generated between the responses and independent variables using the statistical package SPSS. The equations of the responses represent the quantitative effect of independent variables (X_1_ and X_2_) upon the responses (Y_1_, Y_2_ and Y_3_). Coefficients with more than one factor represent the interaction between factors while coefficients with second order terms indicate the quadratic nature of the phenomena.

The equation of the Y_1_ (percent of incorporation efficiency) is given below:

2 $$ {\mathrm{Y}}_1 = 20.560 + 6.101\ {\mathrm{X}}_1\hbox{--}\ 0.{{126\ \mathrm{X}}_1}^2\hbox{--}\ 0.0{{16\ \mathrm{X}}_2}^2 $$

**Fig. 1:**
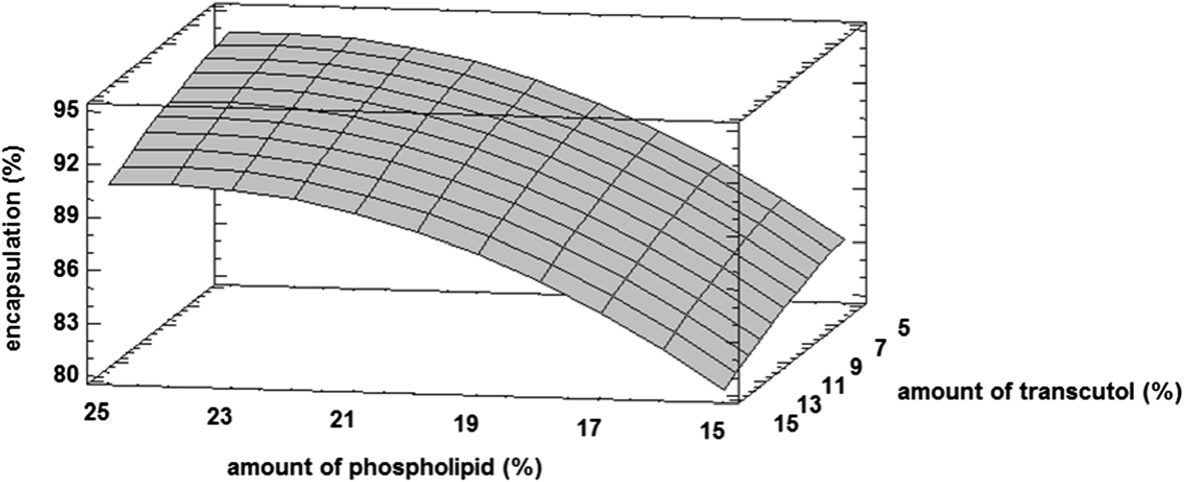
Response surface plot for Y_1_ response (percent of incorporation efficiency)

Three-dimensional response surface plot for Y_1_ is shown in Fig. 1. According to the Fig. 1, incorporation efficiency was increased by increasing the amount of phospholipid, while it was slightly decreased with higher amounts of transcutol. The lipophilic nature of tretinoin may lead to incorporation of this drug between the lipid bilayers. This can also explain the high incorporation efficiency of the tretinoin in liposomal formulations [22].

### In vitro drug release

**Fig. 2:**
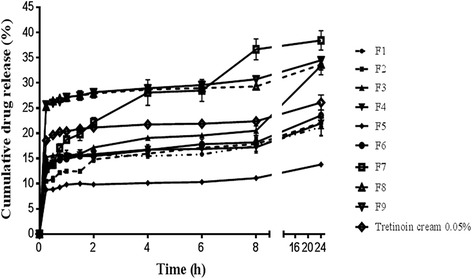
Release profiles of tretinoin from various liposomal formulations and tretinoin cream

The release profile of tretinoin from various liposomal formulations and conventional tretinoin cream are shown in Fig. 2. In the first 2 h, drug release from F8 and F9 were significantly (*P* < 0.05) higher than other formulations, at the 4^th^ hour no statistical significant differences were observed among F7, F8 and F9 (*P* > 0.05) and after 8 and 24 h, F7 showed maximum drug release compared with other formulations. The release pattern of tretinoin cream and all liposomal formulations except F7, showed burst release in initial times and then had a reduced rate of release [23].

The target of optimization was to obtain formulations with slow release in first 15 min and maximum release at 24 h. Therefore constraints for the Y_2_ (percent of drug release in 15 min) and Y_3_ (percent of drug release in 24 h) were:

$$ {\mathrm{Y}}_2 < 15\ \%\ \mathrm{and}\ {\mathrm{Y}}_3 > 30\ \% $$

The equations of the responses Y_2_ and Y_3_ are given below:

3 $$ {\mathrm{Y}}_2 = 101.07\ \hbox{--}\ 9.207\ {\mathrm{X}}_1\hbox{--}\ 1.666\ {\mathrm{X}}_2 + 0.111\ {\mathrm{X}}_1{\mathrm{X}}_2 + 0.{{223\ \mathrm{X}}_1}^2 $$

4 $$ {\mathrm{Y}}_3 = 149.821\ \hbox{--}\ 14.491\ {\mathrm{X}}_1\hbox{--}\ 0.177\ {\mathrm{X}}_1{\mathrm{X}}_2 + 0.{{431\ \mathrm{X}}_1}^2 + 0.{{194\ \mathrm{X}}_2}^2 $$

Analysis of variance (ANOVA) (Table 4) demonstrated that the mathematical models generated were statistically significant and valid for each considered response.

**Table 4:** Analysis of variance (ANOVA) of dependent variables (percent of incorporation efficiency (Y_1_) and percent of drug release in 15 min (Y_2_) and 24 h (Y_3_))

Source of variation	Sum of squares	Degree of freedom	Mean square	*F* ratio	*P*-value
Y_1_					
Regression	604.968	3	201.656	87.409	0.000
Residuals	53.062	23	2.307		
Total	658.030	26			
R^2^ = 0.919					
Y_2_					
Regression	716.867	4	179.217	23.780	0.000
Residuals	165.800	22	7.536		
Total	882.666	26			
R^2^ = 0.812					
Y_3_					
Regression	1569.312	4	392.328	87.047	0.000
Residuals	99.156	22	4.507		
Total	1668.468	26			
R^2^ = 0.941					

**Fig. 3:**
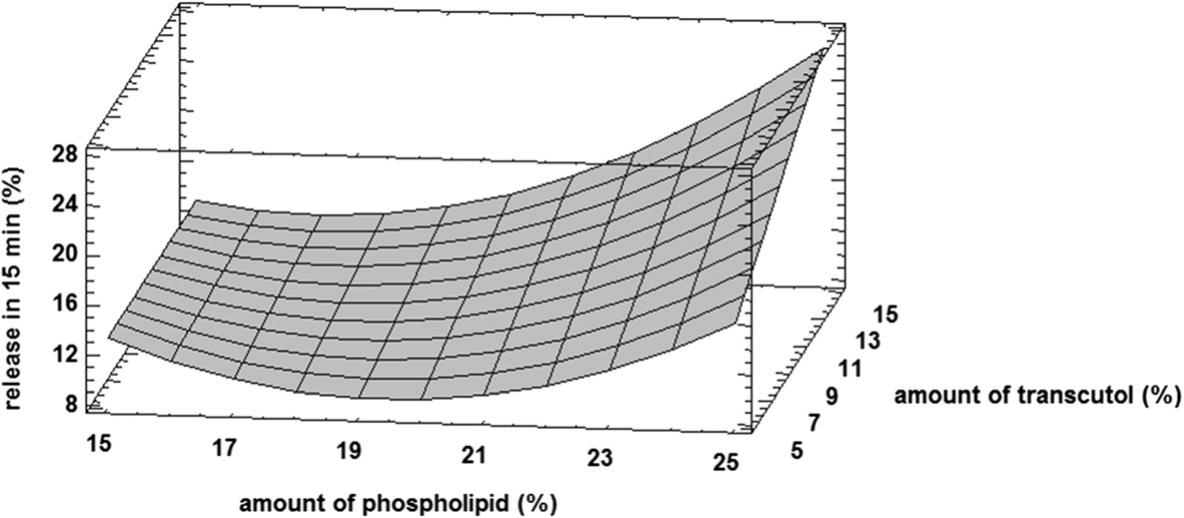
Response surface plot for Y_2_ Response (percent of drug release in 15 min)

**Fig. 4:**
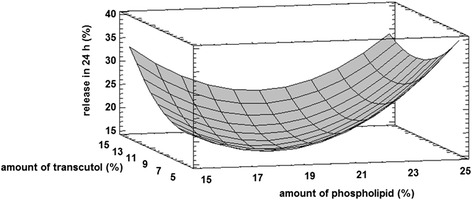
Response surface plot for Y_3_ Response (percent of drug release in 24 h)

Three-dimensional response surface plot for Y_2_ and Y_3_ are shown in Figs. 3 and 4. As shown in Fig. 3, drug release was decreased in 15 min by increase in the concentrations of phospholipids up to 20 %. Meanwhile, increase in the amount of phospholipid from 20 to 25 %, higher the value of drug release. Also it was shown that formulations with higher amounts of transcutol in their compositions released more elements of drug at 15 min.

According to Fig. 4, liposomes containing about 17–19 % phospholipid exhibited the lowest drug release among the formulations. However, it was increased by either lower or higher phospholipid concentrations so that drug release at 24 h from formulations composed of 25 % phospholipid (F7, F8 and F9) was maximums.

The phase transition temperature (T_m_) of the lipids affected the release of liposome [24]. Phospholipids at their T_m_ changed from solid to liquid state. Thus the permeability of liposomal membrane is increased and encapsulated drug released [25]. Chen et al. [26] prepared stealth liposome with different phosphatidylcholine and found liposome composed SPC in the presence of rat plasma showed maximum drug release which due to lower T_m_ of SPC.

**Fig. 5:**
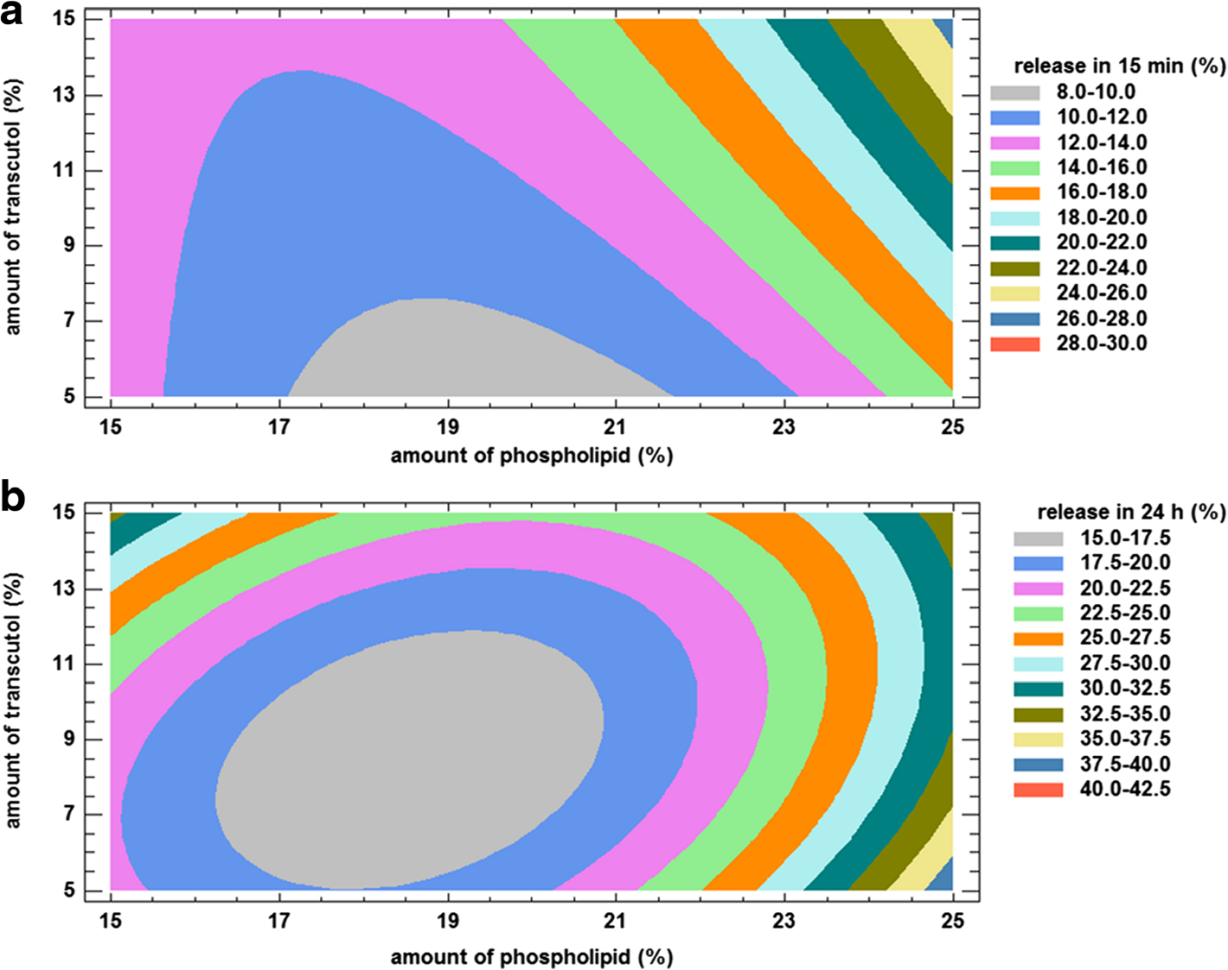
Contour plots for Y_2_ Response (percent of drug release in 15 min) **a** and Y_3_ (percent of drug release in 24 h) **b**

According to contour plots of responses Y_2_ and Y_3_ (Fig. 5), optimized formulations were selected. The defined desirable areas of responses Y_2_ and Y_3_ were superimposed and the region of interest was found. The ratio of SPC: transcutol to obtained optimum formulations were 15.5:14.5, 24:7 and 25:5.

As a validation method for the process, liposomal formulations were prepared at the predicted levels of the independent variables and evaluated for percent of drug release in 15 min and 24 h. Observed and predicted responses for optimum formulations were then compared and the results were shown in Table 5.

**Table 5:** Observed and predicted responses (percent of drug release in 15 min (Y_2_) and 24 h (Y_3_)) for optimum formulations

Formulation (SPC: transcutol)	Dependent variables	Observed response	Predicted response	Residual
F (15.5:14.5)	Y_2_	14.88	12.72	2.61
	Y_3_	30.41	29.76	0.65
F (24:7)	Y_2_	14.88	15.53	−0.65
	Y_3_	33.28	30.06	3.22
F (25:5)	Y_2_	12.97	15.81	- 2.84
	Y_3_	38.43	39.64	- 1.21

Viscosity of F (25:5) and F (15.5:14.5) were 354.53 ± 4.51 and 266.94 ± 3.17 poise, respectively. Formulation (25:5) with the highest amount of phospholipid and lowest amount of transcutol had more viscosity compared with F (15.5:14.5). Thus Formulation (25:5) showed slower release in initial times. Fetih et al. [27] developed and evaluated liposomal gels of celecoxib and concluded that inverse relationship presented between viscosity of liposomal gels with drug diffusion rate and percent of drug released.

According to the results presented in Table 5, observed responses were close to predicted ones which confirmed that the factorial design was valid for predicting the optimum formulation.

### In vitro skin penetration and retention

**Fig. 6:**
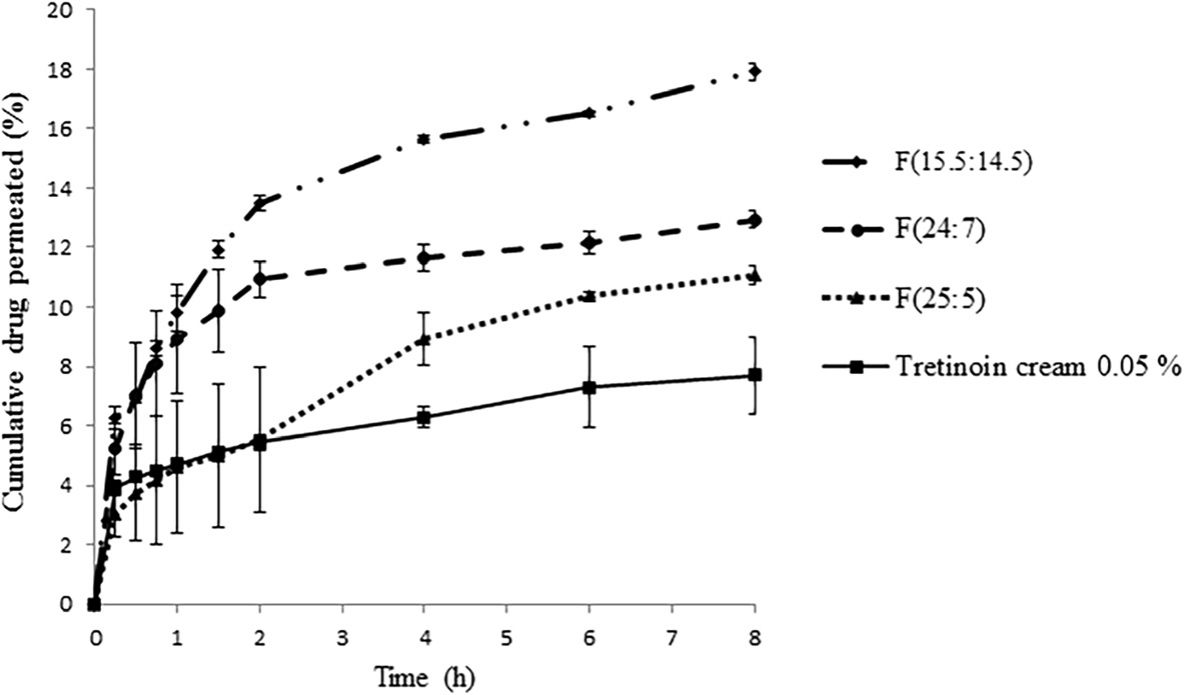
In vitro permeation profiles of tretinoin from cream and optimum formulations through mice skin

The skin penetration profile of F (15.5:14.5), F (24:7), F (25:5) and conventional tretinoin cream are shown in Fig. 6. The penetration percent of optimum formulations was higher (*P* < 0.05) than tretinoin cream. F (15.5:14.5) showed maximum percent of penetration.

According to the Table 6, F (15.5:14.5) showed maximum flux. Formulations and cream penetrated through the skin without any lag time. The flux of F (15.5:14.5) and F (24:7) were higher than cream (*P* < 0.05), while no statistical significant differences found between F (25:5) and tretinoin cream (*P* > 0.05).

**Table 6:** Permeation parameters, Retention (%) and ratio of retention/penetration of tretinoin from optimum formulations and cream (mean ± SD, n = 3)

Formulation	J_ss_ (μg/cm^2^.h)	T_lag_ (h)	Retention (%)	Retention/Penetration
F(15.5:14.5)	4.71 ± 1	0	12.97 ± 2	0.72 ± 0.1
F(24:7)	3.29 ± 0.08	0	8.27 ± 1.91	0.63 ± 0.15
F(25:5)	1.05 ± 0.25	0	13.19 ± 0.71	1.18 ± 0.07
Tretinoin cream	0.88 ± 0.04	0	13.15 ± 2.77	1.72 ± 0.44

ANOVA analysis showed statistical significant differences between J_ss_ of optimum Formulations (*P* < 0.05). These results showed that by enhancement of transcutol concentration in the optimum formulations, percent of penetration and flux was increased which can be due to solubilizing properties of transcutol [28].

The percent of retained tretinoin and ratio of retained drug in the skin to penetrated drug for optimum formulations and tretinoin cream calculated and results showed in Table 6. There was no significant difference in the percent of retained tretinoin between optimum formulations and tretinoin cream (*P* > 0.05). ANOVA analysis showed that no significant difference among ratio of retention/penetration of optimum formulations (*P* > 0.05) but tretinoin cream showed higher ratio of retention/penetration than F(15.5:14.5) and F(24:7) (*P* < 0.05) that could be attributed to higher flux in formulations containing higher amount of transcutol.

Manconi et al. [29] evaluated PEVs with different penetration enhancers for dermal delivery of tretinoin. Their results showed PEVs improved cutaneous drug accumulation compared to control liposome. However, PEVs containing transcutol and labrasol had lower drug accumulated/drug permeated ratio because these vesicles increased both tretinoin deposition and flux.

Mura et al. [30] prepared PEVs with labrasol, transcutol and cineole for (trans) dermal delivery of minoxidil. They concluded that PEVs improved drug deposition into the skin when compared to the classic liposomes.

Caddeo et al. [31] developed PEVs with transcutol or propylene glycol, liposomes and ethosomes for delivered the diclofenac to the skin. Results showed PEVs containing transcutol led to higher drug accumulation in the skin compared to other carriers, diclofenac solution and voltaren. Also they found PEVs containing transcutol permeated the drug more than other vesicular formulations.

### Histological evaluation

**Fig. 7:**
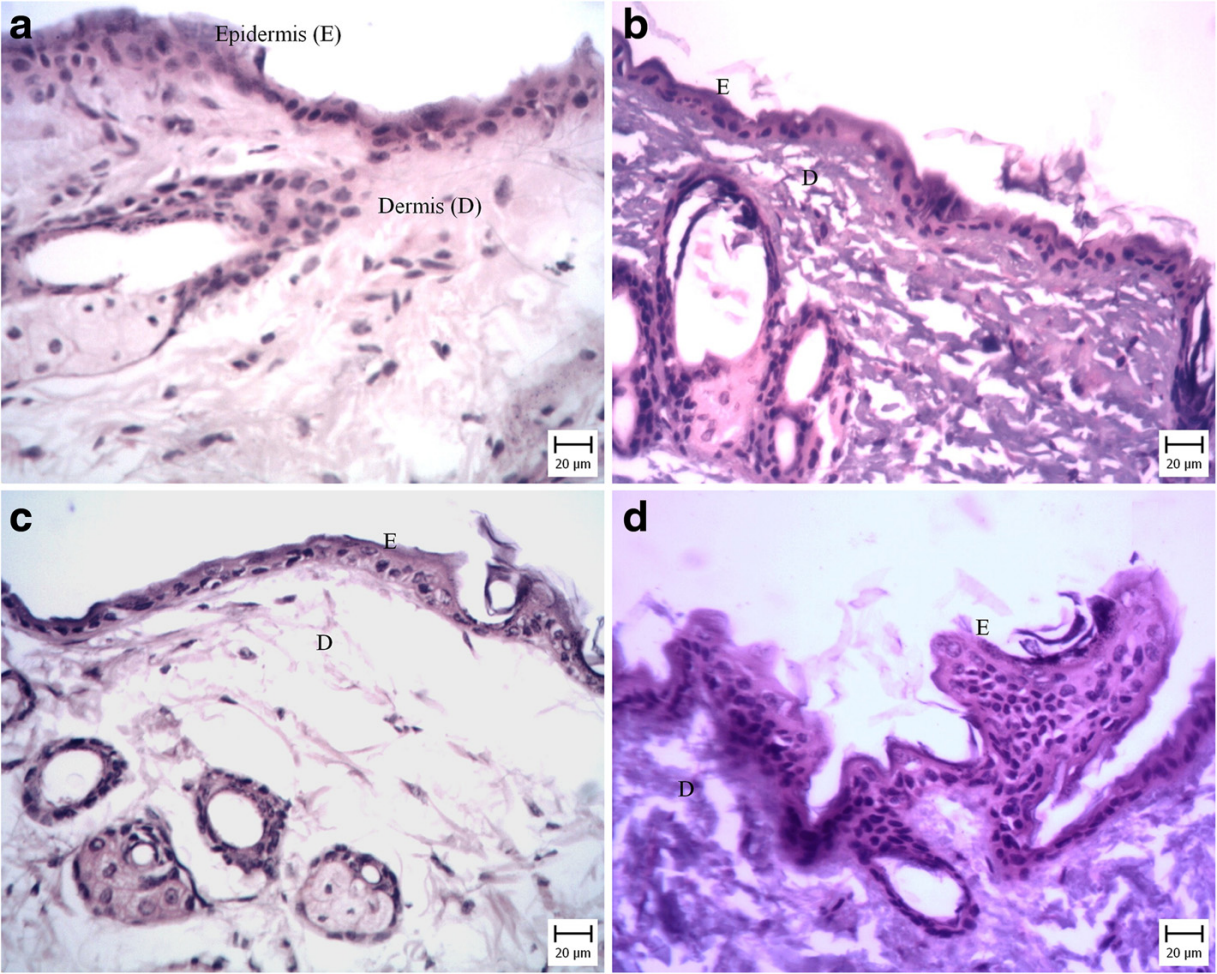
Photomicrographs (20×) of hairless mice skin sections after haematoxylin and eosin staining: **a** untreated skin, **b** skin treated with F(15.5:14.5), **c** skin treated with F(25:5) and **d** skin treated with tretinoin cream 0.05 %

Histopathological investigation revealed that the thickness of epidermis in skin samples treated with F (15.5:14.5) (Fig. 7b) and F (25:5) (Fig. 7c) was same as untreated skin (control group) (Fig. 7a) and hyperkeratosis was increased in comparison with control group. In tretinoin cream treated skin, proliferations of keratinocytes were obvious and also hyperkeratosis was evident (Fig. 7d). Similar results obtained by Ascenso et al. [32]. They evaluated in vivo skin irritation potential of tretinoin loaded ultradeformable vesicles in comparison to ketrel® and demonstrated ultradeformable vesicles caused lowest skin irritation. Their histological study showed in the skin treated with tretinoin loaded ultradeformable vesicle, hyperkeratosis was occurred and for ketrel® treated skin hyperplasia was observed.

Raza et al. [1] developed lipid-based nanocariers for dermal delivery of tretinoin and concluded these carriers were well-tolerated on mouse skin while in the skin treated with marketed product inflammation was observed.

Castro et al. [33] prepared retinoic acid loaded solid lipid nanoparticles and found this cariers considerably reduced skin irritation in rabbit and rhino mouse models when compared to marketed cream. Furthermore their histological evaluation showed in skin treated with retinoic acid loaded solid lipid nanoparticles and marketed gel epidermal thickness was increased in comparison to placebo group.

### DSC

**Fig. 8:**
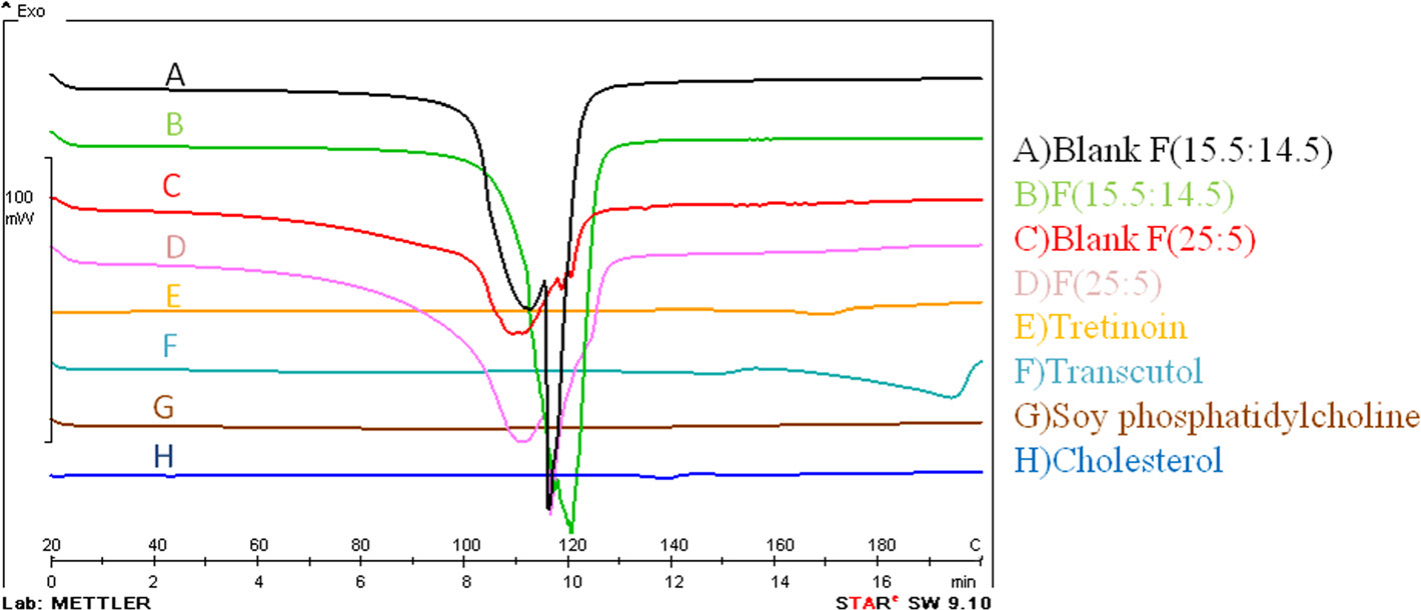
DSC thermograms of **A** Blank F(15.5:14.5), **B** F(15.5:14.5), **C** Blank F(25:5), **D** F(25:5), **E** Tretinoin, **F** Transcutol, **G** Soy phosphatidylcholine, **H** Cholesterol

According to Fig. 8, DSC thermogram of tretinoin and cholesterol showed endothermic peak at 170 °C and 140 °C corresponding to their melting points, respectively [34, 35]. Transcutol thermogram showed endothermic peak about 195 °C, indicating its boiling point [36]. SPC thermogram showed a broad peak at 88 °C. In case of tretinoin loaded liposomes, the melting point of tretinoin was not observed which indicates that it is encapsulated in the liposome. The blank and tretinoin loaded liposome showed peak around 100–120 °C in their thermograms, which may be caused by evaporation of bounded water.

### Stability study

**Fig. 9:**
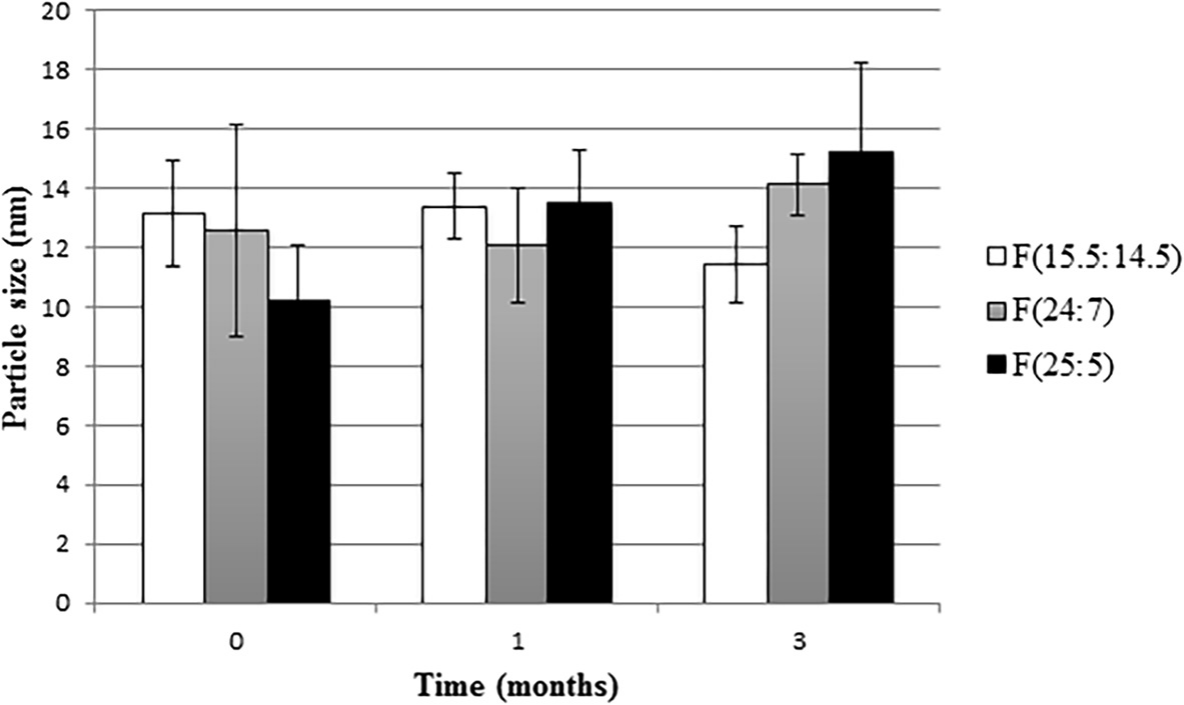
Mean particle size (nm) of optimum formulations after 1 and 3 months storage at 4 °C

**Fig. 10:**
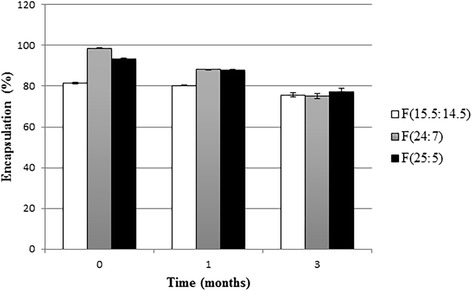
Incorporation efficiency (%) of optimum formulations after 1 and 3 months storage at 4 °C

The results of stability study for optimum formulations are shown in Figs. 9 and 10. Over the course of 3 months, the mean particle size of formulations did not change significantly (*P* > 0.05). This matter could be resulted by the presence of transcutol in the formulation that may cause flexibility in vesicles [30] and cholesterol that has stabilizing effect against aggregation and fusion of the liposomes [37]. Similar results obtained by Mura et al. [38] that found particle size of PEVs with different amounts of transcutol remained constant during 3 months at 4 °C. While, particle size of liposomes without transcutol increased significantly. Incorporation efficiency of F (24:7) and F (25:5) were significantly decreased (*P* < 0.05) after 1 and 3 months while drug loading of F (15.5:14.5) only decreased about 6 % after 3 months (*P* < 0.05).

Chessa et al. [39] evaluated influence of PEVs with different types of penetration enhancer on (trans) dermal delivery of quercetin and concluded all PEVs promoted drug deposition in the skin.

Srisuk et al. [40] prepared deformable liposomes containing oleic acid for transepidermal delivery of methotrexate. These vesicles showed the highest skin permeation, accumulation and flux compared to liposomes without oleic acid and methotrexate solution.

Charoenputtakun et al. [41] developed solid lipid nanoparticles, nanostructured lipid carriers and nanoemulsions containing limonene or cineol as penetration enhancer for dermal delivery of all-trans retinoic acid. They demonstrated solid lipid nanoparticles containing limonene had the highest skin permeation and flux in compression to other formulations and all-trans retinoic acid suspension.

## Conclusion

PEVs are novel class of liposomes which consist of phospholipid and penetration enhancer. In this work deformable liposome containing SPC and transcutol was employed for dermal delivery of tretinoin. Particle size of all formulations was smaller than 20 nm. Liposomes showed high incorporation efficiency which may be due to lipophilic nature of tretinoin. Formulations containing 25 % phospholipid exhibited the highest drug release at 24 h while the amount of transcutol did not significantly change drug release. Drug penetrated through the skin for optimum formulations was higher than cream which can be due to solubilizing properties of transcutol. Optimum formulations compared to tretinoin cream caused milder hyperkeratosis without hyperplasia. These results suggested that deformable liposomes could gradually released tretinoin and thus decreased its adverse effects such as erythema, peeling, burning and also increased patient compliance.

## Abbreviations

DSC: Differential Scanning Calorimetry
Jss: Steady-state permeation rate
PEV: Penetration-Enhancer Vesicle
SPC: Soy Phosphatidylcholine
